# Brown and White Sugars

**Published:** 1921-02-05

**Authors:** 


### Brown and White Sugars.

Some interesting points in connection with the ordinary use of brown and white sugars are raised in a recent note in the *Lancet* by M. C. Soar, B.Sc., F.C.S., who compares the values of samples obtained indiscriminately from ordinary commercial sources. Apparently the colour produced is the only serious disadvantage in the employment of brown sugar for cooking. Out of six samples, varying from yellow to a dark brown, only two contained less than 90 per cent. of cane sugar; and if invert sugar were included in the figure, the average for all samples was fully 93 per cent.

For persistence of the sweet taste upon the palate the presence of invert sugar is actually an advantage. Deficiency in cane sugar causes de'ay in its first appreciation. In this respect, therefore, the defect of brown sugar appears to arise simply with the fact that in sweetening the white sugar acts more quickly. None of the brown samples contained any albuminoid matter, and the ash left after burning was in every case less than 1 per cent., so that no animal matter and no sand adulterant could have been present. No dyes and no excess of moisture could be detected, the only impurities being simply those vegetable substances natural to raw sugars. These tests should certainly tend to dispose of some of the popular prejudice against the various coloured varieties of sugar.

